# Multiple homoplasious insertions and deletions of a Triticeae (Poaceae) DNA transposon: a phylogenetic perspective

**DOI:** 10.1186/1471-2148-7-92

**Published:** 2007-06-14

**Authors:** Roberta J Mason-Gamer

**Affiliations:** 1 Department of Biological Sciences, University of Illinois at Chicago, M/C 066, Chicago, Illinois, USA

## Abstract

**Background:**

*Stowaway *elements are short, non-autonomous DNA transposons categorized as miniature inverted-repeat transposable elements (MITEs). The high MITE copy number in grass genomes suggests an active history of amplification and insertion, but ongoing MITE activity has only rarely been seen, and ongoing *Stowaway *activity has never been observed. Thus, a phylogenetic perspective on presence vs. absence of elements in an aligned data set can provide valuable historical insights into the dynamics of MITE acquisition and loss.

**Results:**

A *Stowaway*-like element resides within the fourth intron of a β-amylase gene in representatives of five genera in the wheat tribe, Triticeae. Its presence vs. absence was examined with reference to the β-amylase gene tree topology, and in light of sequence comparisons of the β-amylase elements to Triticeae *Stowaway *elements in the Entrez nucleotide database. Among the sequences lacking the element, there are five distinct putative excision footprints (one widespread and four restricted to unrelated lineages) and two flanking deletions. The sequences that do contain elements are polyphyletic on the β-amylase tree, and their elements are divergent at the sequence level. The β-amylase elements do not form a monophyletic group relative to other *Stowaway *elements in Entrez; most are more similar to elements from other loci in other Triticeae genomes than they are to one another.

**Conclusion:**

Combined, the phylogenetic distribution, sequence variation, and Entrez database comparisons indicate that a *Stowaway*-like element has undergone multiple deletions from and insertions into the same site in β-amylase intron 4 during the history of the tribe. The elements currently at the site represent multiple, distinct lineages that transcend generic boundaries. While patterns of *Stowaway *polymorphism across a phylogenetic data set do not allow evolutionary mechanisms to be inferred with certainty, they do provide insights into the dynamics of element evolution over an extended time scale. The historical perspective provided by a phylogenetic approach is complementary to the few studies in which ongoing MITE activity has been documented.

## Background

Transposable elements (TEs) are divided into two main classes depending on their mode of transposition [1]: class I elements transpose through an RNA intermediate using a reverse transcriptase, while class II elements transpose through a DNA intermediate. Class II elements are further classified as either autonomous elements, which include a gene encoding a transposase, or non-autonomous elements, which do not encode a functional transposase. Miniature inverted-repeat (IR) transposable elements (MITEs) are members of a highly repetitive category of class II non-autonomous elements, and are extremely abundant in plant genomes [2-4]. These small (<500 bp) elements are recognized by their by short terminal inverted repeats (TIRs), and 2–3 bp target site duplications (TSDs).

MITEs are further grouped into families based on differences among their TIR sequences and TSDs, and on their relationships to known autonomous elements from which the families appear to have been derived [5]. The subset of MITEs categorized as *Stowaway *elements [6] share similar 10-bp TIRs (consensus CTCCCTCCRT), a 2-bp target site preference (5'-TA-3'), and the potential to form secondary structures [7,8]. Transposition of *Stowaway *elements is hypothesized to be associated with autonomous *mariner*-like elements based on their TIR similarities [8], on the discovery of *Stowaway *elements with open reading frames that share sequence similarity with known *mariner *transposases [9-11], and on the observed interaction between *mariner*-like transposases and *Stowaway *MITEs in rice [12].

Because very few MITEs have been observed to be actively transposing, the dynamics underlying their gain, loss, and high copy number are not well understood [5]. Aligned sequence data, however, can help clarify certain aspects of MITE evolution over a longer time scale [13]. The present study uses phylogenetic information to analyze patterns of sequence diversity and presence vs. absence of *Stowaway*-like elements in the fourth intron of a β-amylase gene, using aligned sequences from a broadly representative sample of the wheat tribe, Triticeae. Two main observations reveal a complex history of multiple losses and multiple gains of the element. First, the sequence variation adjacent to the empty sites, and its phylogenetic distribution, suggests numerous independent losses. There are five distinct putative excision footprints: one that is widespread throughout the sample, and four that are restricted to unrelated lineages. Two additional empty sites, in unrelated sequences, are associated with deletions flanking intron sequence. Second, the occurrence of divergent elements at the same site in the gene, and their phylogenetic distribution, suggest multiple acquisitions of elements at the site. The sequences that have elements, representing five Triticeae genera, are polyphyletic on the β-amylase gene tree. More strikingly, four of the five β-amylase elements show greater similarity to elements in other genes and in other Triticeae genomes than they do to one another; i.e., the elements at this site do not form a monophyletic group relative to other known *Stowaway *elements. Taken together, these sequence comparisons highlight the complex evolutionary history of *Stowaway*-like elements at this locus.

## Results

**Figure 1:**
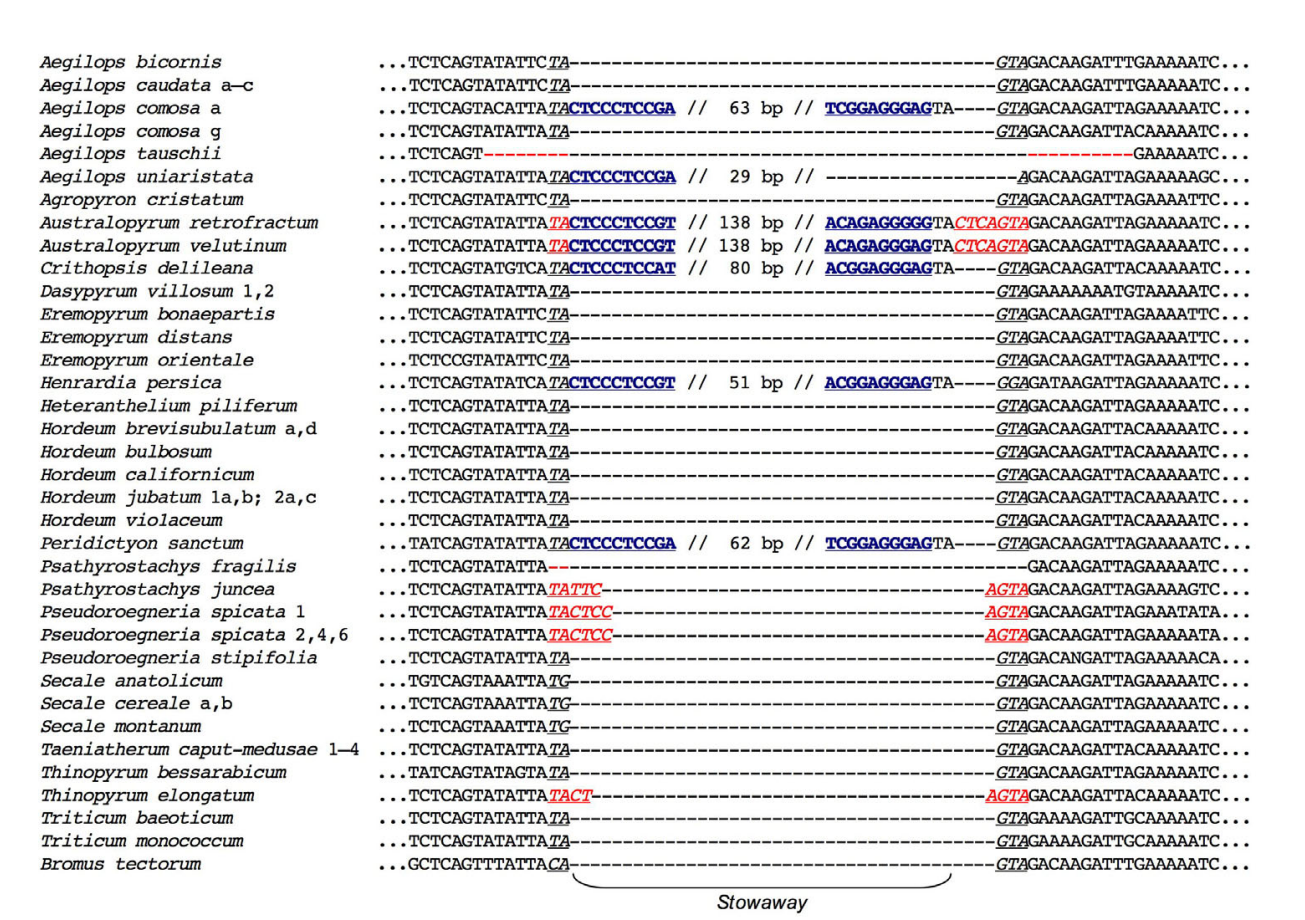
**Alignment of the region of β-amylase intron 4 containing *Stowaway *elements**. The 10-bp terminal repeats are shown in underlined blue boldface. A widespread putative excision footprint is shown in underlined black italics; other hypothesized footprints are shown in underlined red italics. The lengths of the internal portions of the elements are given within the double slashes. *Ae. uniaristata *contains a partial element similar to the 5' end of the element from *Ae. comosa*. Where relevant, numbers after taxon names distinguish individuals within species, and letters distinguish cloned sequences from within individuals.

Short, palindromic insertions were found in β-amylase intron 4 in representatives of five Triticeae genera (Fig. 1). They appear to be *Stowaway *elements, based on their size, their conserved 10-bp terminal repeats, their palindromic structure, and their TA insertion site [7]. From sequence information alone, however, it is impossible to determine whether these specific palindromic structures behave like true *Stowaway *elements, i.e., whether they have the potential to be excised by *mariner*-like transposases, or whether they occur in the very high copy numbers characteristic of *Stowaway *elements. Based on results of searches of the Entrez nucleotide database (see below), the five elements vary in their frequency of occurrence at non-homologous sites in Triticeae genomes, yielding between 3 and 81 hits in the database. However, while *Stowaway *elements are collectively numerous, there is no reason to assume that every *Stowaway *variant will have a very high copy number. This is a highly dynamic group of rapidly-evolving sequences, and their history (in rice, at least) appears to have involved several temporally-separated waves of amplification [8]. Thus, abundances of different *Stowaway *variants are expected to vary widely (and change through time).

**Figure 2:**
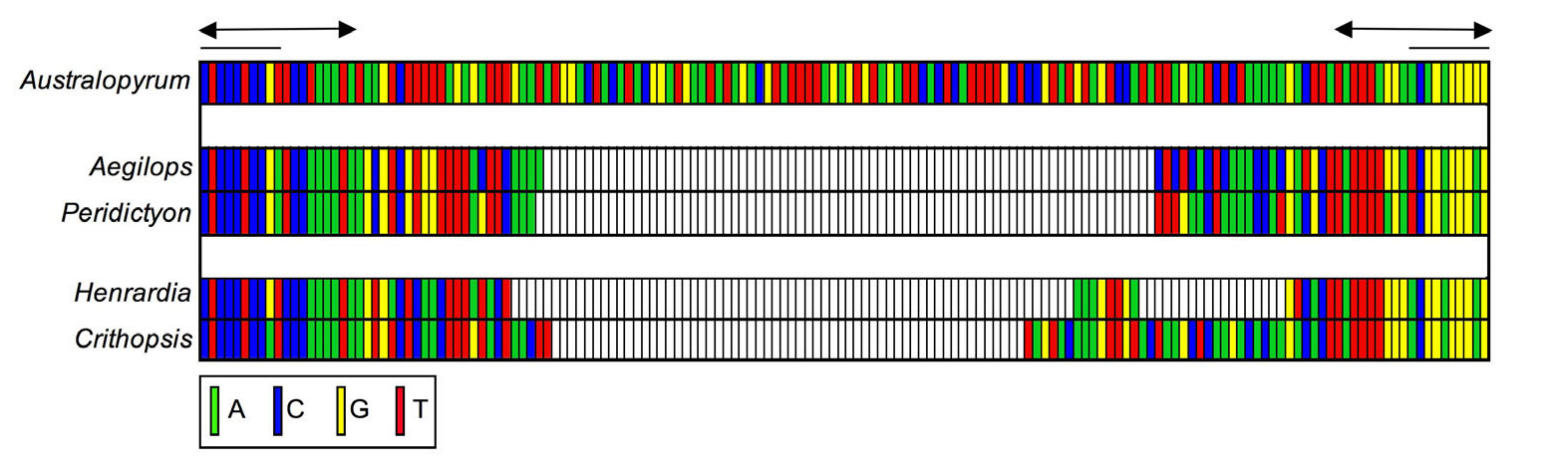
**Alignment of the β-amylase intron 4 *Stowaway *elements**. The terminal portions (double arrows) of all five elements are alignable, but straightforward alignments of the central portions are possible only between *Aegilops *and *Peridictyon*, and between *Henrardia *and *Crithopsis*. Plain lines above the alignment correspond to the 10-bp TIRs.

Although the characteristic TIRs and TSDs of the five β-amylase elements are easy to align, the regions between the TIRs (excluded from Fig. 1 alignment) are variable in both length and sequence (Fig. 2). *Australopyrum retrofractum *and *Au. velutinum *have nearly identical 158-bp elements (98.7% sequence identity), *Aegilops comosa *contains an 83-bp element, *Peridictyon sanctum *has an 82-bp element, *Henrardia persica *has a 71-bp element, and *Crithopsis delileana *contains a 100-bp element. (*Aegilops uniaristata *contains a 38-bp partially degraded element that is 94.7% similar to the 5' end of the *Ae. comosa *element.) The *Ae. comosa *and *P. sanctum *elements are easy to align (1 bp length difference; 89% identity), as are the *C. delileana *and *H. persica *elements, though these differ in length (29 bp length difference, 94.4% identity excluding gaps; Fig. 2). Other pairwise combinations of the β-amylase elements cannot be unambiguously aligned (Fig. 2).

Most of the β-amylase sequences show evidence of element excision and/or degradation, including several distinct footprints that are consistent with the proposed mechanism of excision of a rice *Tc1*/*mariner *element in an experimental yeast system [14]. Nearly all of the empty sites, and all of the element-containing sites, are flanked by TA...GTA (Fig. 1, black italics). In addition to this widespread footprint, sequences representing four genera have distinct excision footprints consisting of short TIR fragments (Fig. 1, red italics). These flank empty sites in *Pseudoroegneria spicata *(footprint TACTCC...AGTA); *Psathyrostachys juncea *(TATCC...AGTA); *Thinopyrum elongatum *(TACT...AGTA); and a now-occupied site in *Australopyrum retrofractum *and *Au. velutinum *(TACTC...AGTA). Finally, two sequences lack the element along with portions of the intron flanking the empty site (Fig. 1), including *Aegilops tauschii *(missing 6 bp 5' and 10 bp 3' of the putative insertion site), and *Psathyrostachys fragilis *(missing only the original TA insertion site). It is not clear whether the deletions are associated with element loss.

**Figure 3:**
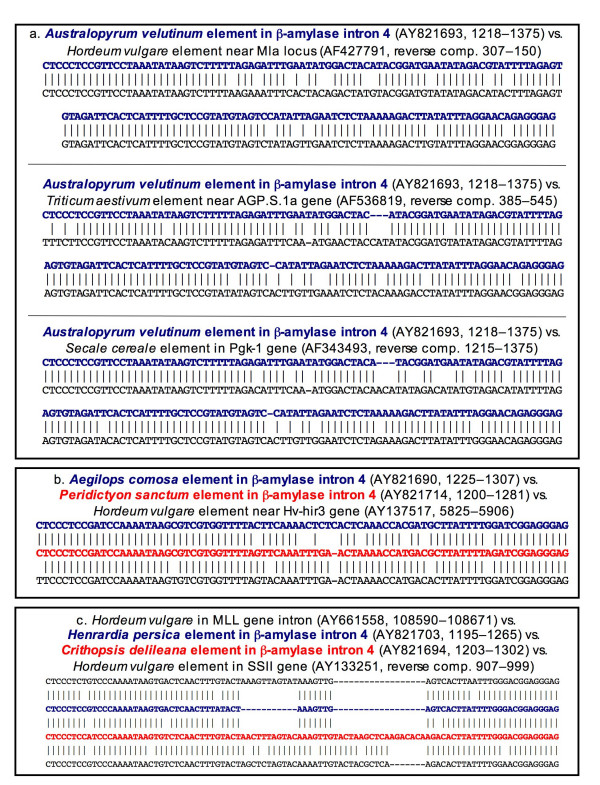
**BLAST search results**. BLAST matches (black font) to each of the five distinct elements in β-amylase intron 4 (red or blue boldface font). a. Numerous close matches were found to the *Australopyrum *element; those shown are within or near three different loci in three different Triticeae genera: a powdery mildew resistance locus in *H. vulgare*; an ADP glucose phosphorylase gene in *T. aestivum*; and a 3-phosphoglycerate kinase gene in *S. cereale*. b. The closest match to the *Ae. comosa *β-amylase element (blue) is the *P. sanctum *β-amylase element (red); the closest match to the *P. sanctum *element is near a *Hordeum *hypersensitive-induced reaction protein gene (black). c. The *H. persica *(blue) and *C. delileana *(red) β-amylase elements are similar in sequence but differ in length by 29 bp. The *H. persica *element is more similar in both length and sequence to a *Hordeum *element near a putative RNA binding protein gene (black, top line); the *C. delileana *β-amylase element is more similar in length, though slightly less similar in sequence, to an element in a *Hordeum *starch synthase II gene (black, bottom line). Table 1 provides details about the length and sequence comparisons shown here.

Although the β-amylase elements differ from one another, BLAST searches of the Entrez nucleotide database reveal that each shows marked similarity to one or more putative *Stowaway *elements at other loci in other Triticeae genomes (Table 1). The *Australopyrum *elements yield 81 close matches (with "close match" defined here as a sequence that covers 85–100% of the query sequence with 85–100% identity); this is the largest number of close matches obtained. These are found at multiple loci in the genomes of barley, wheat, rye, and in two diploid wheat progenitors, thus illustrating the broad distribution of this particular element type throughout Triticeae genomes. Three of these are shown (Fig. 3a, Table 1), including one near a powdery mildew resistance locus (M1a) in barley (same length, 89.2% identity); one near an ADP-glucose pyrophosphorylase gene (AGPase) in wheat (4 bp length difference, 89.9% identity); and one near a phosphoglycerate kinase gene (Pgk1) in rye (4 bp length difference, 89.9% identity). Similar elements were also found in some diploid wheat relatives (not shown), including: *Triticum monococcum *(AY951945, reverse complement 23114–23270; 1 bp length difference, 87.3% identity) and *Aegilops tauschii *(AY534123, 69955–70115, 4 bp length difference, 90.4% identity). Of only three close matches to the *Ae. comosa *β-amylase element, the closest is the *P. sanctum *β-amylase element (1 bp length difference, 89% identity), but the closest match (out of six) to the *P. sanctum *element is from barley, near a hypersensitive-induced response (HIR) gene (equal in length and 93.9% identity; Fig. 3b, Table 1). Of the 22 close matches to the *H. persica *element, one of the closest is in a barley gene for a putative RNA binding protein (1 bp length difference, 95.8% identity; Fig. 3c, Table 1). The *C. delileana *element is 95% identical to the *H. persica *element (Fig. 3c, Table 1), but is a full 29 bp longer. Because of the length difference, it is not among the 14 closest matches as defined above; one of these is near a barley starch synthase (SSII) gene (7 bp length difference, 92.5% identity; Fig. 3c, Table 1).

**Table 1:** BLAST search results for β-amylase *Stowaway*-like elements; see Fig. 4 for alignments.

β-amylase element	BLAST matches to β-amylase elements^1^					
Species, Genbank Accession, Range	Genbank Accession, Range	Genome	Location	E-value^2^	Length Difference	Sequence Similarity^3^

*Australopyrum velutinum * AY821693, 1218–1375	AF427791 307–150	*Hordeum vulgare*	Powdery mildew resistance locus	3 × 10^-42^	0	89.2% (141/158)
*Australopyrum velutinum * AY821693, 1218–1375	AF536819 545-385	*Triticum aestivum*	ADP glucose phosphorylase gene	1 × 10^-35^	4	89.9% (142/158)
*Australopyrum velutinum * AY821693, 1218–1375	AF343493 1375-1215	*Secale cereale*	3-phosphoglycerate kinase gene	2 × 10^-31^	4	89.9% (142/158)
*Aegilops comosa * AY821690, 1225–1307	AY821714 1200–1281	*Peridictyon sanctum*	β-amylase gene	4 × 10^-12^	0	89.0% (72/82)
*Aegilops comosa * AY821690, 1225–1307	AY137517 5906-5825	*Hordeum vulgare*	Hypersensitive-induced reaction protein gene	2 × 10^-11^	1	86.6% (71/82)
*Peridictyon sanctum * AY821714, 1200–1281	AY137517 5825–5906	*Hordeum vulgare*	Hypersensitive-induced reaction protein gene	1 × 10^-27^	1	93.9% (77/82)
*Henrardia persica * AY821703, 1195–1265	AY661558 108590–108671	*Hordeum vulgare*	Putative RNA binding protein gene	2 × 10^-10^	12	95.8% (68/71)
*Henrardia persica * AY821703, 1195–1265	AY821694 1203–1302	*Crithopsis delileana*	β-amylase gene	1 × 10^-5^	29	94.4% (67/71)
*Crithopsis delileana * AY821694, 1203–1302	AY133251 999-907	*Hordeum vulgare*	Starch synthase II gene	6 × 10^-18^	7	92.5% (86/93)

^1^In most cases, numerous matches were found (see Results); examples of the closest matches are shown here.

^2^The number of comparable matches expected to be found in the Entrez database by chance alone. Very low E-values can be based on partial matches, and thus do not correspond precisely to sequence similarity.

^3^Gaps are excluded from sequence similarity estimates between elements that differ in length.

**Figure 4:**
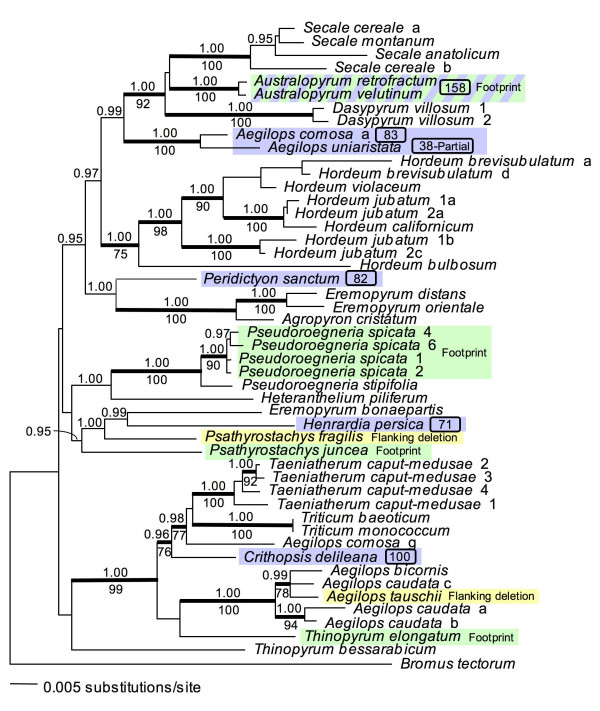
**Phylogenetic distribution of *Stowaway *elements and empty sites**. Maximum-likelihood (ML) estimate of Triticeae relationships based on a previous analysis of β-amylase gene sequences [15], showing the phylogenetic distribution of intron 4 *Stowaway *elements and excision sites. Where relevant, numbers after taxon names distinguish individuals within species, and letters distinguish cloned sequences from within individuals. Bayesian posterior probabilities ≥ 0.95 are shown above nodes, and ML bootstrap support ≥ 75% is shown below nodes. Blue boxes indicate partial and full elements; element lengths are given after the corresponding taxon names. Green boxes indicate sequences with putative excision footprints other than the widespread TA...GTA footprint (Fig. 1). The blue and green box indicates an insertion next to an existing footprint. Yellow boxes indicate sequences lacking the element along with portions of the flanking intron.

The phylogenetic analysis of the β-amylase data set yielded a single ML tree with a score of -lnL = 8846.175 (Fig. 4) [15]. The branching pattern is consistent with the recovery of a single β-amylase homolog, in that most of the genera from which multiple accessions have been sampled form monophyletic groups (*Secale*, *Australopyrum*, *Dasypyrum*, *Hordeum*, *Pseudoroegneria*, and *Taeniatherum*); this pattern is indicative of orthology. Most of the non-monophyletic genera on this tree are non-monophyletic on other gene trees (*Eremopyrum*, *Aegilops*, and *Thinopyrum*) [15]; thus, their non-monophyly on the β-amylase tree does not suggest paralogy. The polyphyletic placement of *H. jubatum *reflects its tetraploid origin. The most unexpected result is the placement of sequences from *Aegilops comosa *and *Ae*. *uniaristata *far from their expected relatives in *Aegilops *and *Triticum*. These outlying copies were not recovered from any other *Aegilops *species, despite repeated attempts. A duplication event deep enough in the tree to explain the two *Aegilops *outliers as paralogs (i.e., a basal duplication) should be apparent in most, if not all, of the genera. Thus, as discussed earlier [15], the *Aegilops *outliers are more consistent with introgression than with ancestral duplication. The interpretation of the β-amylase tree with respect to the evolution of the Triticeae, and relative to other molecular phylogenetic analyses of the tribe, has been discussed [15]; the present paper focuses on its significance with regard to the evolution of the *Stowaway *elements in intron 4.

Sequence variation among empty sites and among complete elements, and their distributions on the β-amylase tree (Fig. 4), suggest a complex history of acquisition and loss. A single early acquisition of an element would explain the broad phylogenetic distribution of footprints and elements throughout the tribe, but would require as many as 11 independent losses to explain the phylogenetic distribution of the widespread TA...GTA footprints, in addition to the four losses leaving distinct footprints (*Pseudoroegneria spicata*, *Psathyrostachys juncea*, *Thinopyrum elongatum*, and *Australopyrum retrofractum *and *Au. velutinum*). A scenario involving multiple insertions into the same site would require as many as five independent acquisitions, or at least three if nodes with low bootstrap support are discounted. If all of the sequences that may have formerly had elements were included (those with putative footprints or deletions of the flanking intron; labeled in Fig. 4), then additional acquisitions (followed by excisions) must be inferred. Note that this scenario would explain only the phylogenetic pattern, and would not account for the sequence differences among the elements, or their similarities to Triticeae *Stowaway *elements at non-orthologous loci.

## Discussion

The utility of phylogenetic information for understanding the evolutionary dynamics of *Stowaway *excision was demonstrated in another example from the Triticeae, which provided evidence for multiple losses of an element from a gene encoding a disrupted meiotic cDNA 1 (DMC1) protein [13]. The present study, based on elements and excision footprints in the fourth intron of a β-amylase gene, provides evidence that both losses and gains have occurred repeatedly in this intron.

In general, multiple independent losses of *Stowaway *elements seem feasible, and even likely. First, the elements are palindromes, and DNA palindromes have long been recognized for their instability. Their tendency to form hairpin structures appears to facilitate slipped-strand mispairing by bringing short, terminal direct repeats into close proximity [16-18]. Thus, their inverted-repeat structure may facilitate their degradation. Second, *Stowaway *elements could be excised during transposition. Although they do not encode a transposase, the resemblance between the TIRs and subterminal sequences of *Stowaway *MITEs and *mariner*-like class II elements [5,8], and the demonstrated physical interaction between *mariner*-like transposases and *Stowaway *MITEs from rice [12], support a mechanism of *Stowaway *excision by a *mariner*-encoded transposase [19]. No *Stowaway *elements have been demonstrated to be currently active, but the DMC1 [13] and β-amylase gene data sets, both of which sample broadly throughout the Triticeae, reveal losses occurring over the time scale of the tribe's history, which by one estimate began between 13 and 25 million years ago [20]. The half-element in *Ae. uniaristata *shows a clear case of degradation of an element similar to the one in *Ae. comosa*. Otherwise, the inferred sites of element loss fall into two categories: (a) five different apparent excision footprints, consisting of 2–4 bp adjacent to the 5' terminal TA duplication, and 1–2 bp adjacent to the 3' TA duplication; and (b) two small deletions of the intron sequence flanking empty element sites.

Because active *Stowaway *excision has not been observed, and MITE excision in general observed only rarely, exactly what a *Stowaway *excision footprint should look like is not known. Transposition footprints from the rice MITE *mPing*, the only group of MITEs observed to be actively transposing, have been characterized in several recent studies [21-24]. However, *mPing *is associated with a different transposase superfamily (*PIF/Harbinger*) than is *Stowaway *(*Tc1/mariner*); thus, *mPing *footprints might not be good predictors of *Stowaway *footprints. Here, inferences are drawn from historical losses of *Stowaway *from a different Triticeae gene [13], and from observed excisions of *Osmar5*, a rice *Tc1*/*mariner *element, in an experimental yeast system [14]. The inferred *Stowaway *footprints from the Triticeae DMC1 gene [13] are similar to those observed in the β-amylase dataset, with 1–4 bp TIR fragments internal to the terminal TA duplications. Like the β-amylase dataset, the DMC1 dataset includes several distinct footprints at orthologous sites within the gene; these were interpreted as evidence of either multiple excisions, or evolution of an ancestral footprint following a single excision event [13]. The putative footprints from both the DMC1 and β-amylase data sets are consistent with the proposed mechanism of excision of the rice *Osmar5 *element in yeast [14], which also yielded footprints with 1–4 bp TIR fragments adjacent to the TA duplications. Whether or not the two β-amylase sequences with deletions adjacent to the empty sites represent additional cases of element loss is not clear, although one case of *Osmar5 *excision from yeast did involve deletions of flanking sequence [14].

While multiple deletions of a transposon are to be expected, there also appear to have been multiple independent insertions of *Stowaway *elements into orthologous sites within intron 4. This would explain their polyphyletic distribution on the β-amylase tree, their sequence diversity, and their marked similarities to elements at non-homologous sites in other genomes. In addition, the *Australopyrum *sequences provide clear evidence of multiple insertions at the same site in the same sequence: the current full element was inserted at the same TA recognition site as an earlier element, which has since been excised leaving a footprint. Transposons do not generally integrate into random sites [25,26]; not only do they insert at specific target site sequences, but available target sites are not selected at random. Some *Oryza *and *Zea *MITEs, for example, are more likely to be found within other MITEs, and they sometimes target specific sites within those MITEs [27]. Extreme specificity, involving multiple insertions into the exact same site in the genome, has been demonstrated for a variety of transposons. Cases involve both class I elements, including SINEs in *Peromyscus *[28] and *ingi *LINEs and RIME SINEs in *Trypanosoma *[29], and class II elements, including a *Pokey *element in *Daphnia *[30], *hobo *elements in *Drosophila *[31], and a striking example of six independent insertions of two distinct *PIF *elements into the same location in an *r*-gene in *Zea *[32]. Site-specific insertions by members of the *Tc1/mariner *transposon family (which includes *Stowaway*) have been associated with features adjacent to preferred TA insertion sites, including the specific sequence immediately flanking the insertion site of *Tc1 *in *Caenorhabditis elegans *[33], and the predicted deformation of DNA at sites of *Sleeping Beauty *insertion during interplasmid transposition in HeLa cells, and into *Mus *sequences [34]. Specific features associated with *Stowaway *insertion site preference in the Triticeae might be clarified by extensive comparisons among the sequences flanking multiple non-homologous insertion sites. No Triticeae species have been fully sequenced, but there is extensive sequence data available from wheat, barley, and rye. Thus, while it would not be possible to carry out as thorough a genome-wide survey of *Stowaway *elements as was recently done for rice [8], the large number of sequenced Triticeae elements in Entrez should provide an excellent starting point for comparisons among *Stowaway *flanking sequences.

One alternative hypothesis to multiple gains is that there was a single gain early in the history of the tribe, and that the observed diversity among the β-amylase elements arose at that site. Given the wide distribution of the TA...GTA excision footprint, there probably was, in fact, an ancestral element at the site, but that element is probably not ancestral to the present elements at the site. First, all of the current elements are themselves enclosed within TA...GTA footprints; thus, the putative earlier element was excised, and did not evolve into the elements now in place. Second, the five current elements are all palindromes, with strong predicted secondary structure. If a single ancestral palindrome has simply evolved very rapidly, the resulting sequences would not maintain any palindromic structure. Similarly, if a large ancestral element were to differentially decay into a series of independently derived smaller elements, the derivatives would not be palindromes. (One feasible exception to this assertion would be a sequential deletion of the central portion of a large palindrome, which would lead to a series of nested palindromes of decreasing size, but this is not the pattern seen here.) The second problem with the single-gain hypothesis cannot be easily dismissed. The similarity of the β-amylase variants to *Stowaway *elements at other loci in other genomes, rather than to one another, strongly refutes the monophyly of the β-amylase elements. Otherwise, whatever process produced the five β-amylase elements from a single ancestor would have to have been operating in parallel at multiple loci in multiple genomes.

The similarity among *Stowaway *elements from different genomes (e.g., *Australopyrum*, *Hordeum*, *Secale*, and *Triticum*; Fig. 3a), and diversity among elements within genomes (e.g., the *Hordeum *elements in Fig. 3), highlight a disconnect between the evolutionary relationships among the elements and the relationships among the species and genera that harbor them. This disconnect is consistent either with the existence of multiple *Stowaway *lineages in the ancestral Triticeae genome, or with horizontal transfer of elements among Triticeae lineages. The observed high level of diversity among the *Stowaway *elements suggests that some of the distinct *Stowaway *lineages are old, and possibly already present in the ancestral Triticeae genome. On the other hand, the close similarity among some of the elements that are shared by phylogenetically distinct genera suggests recent movement of elements among genera. The history of hybridization and introgression among divergent Triticeae lineages [35] would facilitate horizontal transfer, and the spread of *Stowaway *variants among species. Thus, ancestral polymorphism and ongoing introgression may both play a role in the present element diversity, but it is difficult to disentangle these possibilities, especially without a better understanding of how the elements evolve. If, for example, they evolve extremely quickly, the suggestion that the lineages are old based on their high level of divergence is not valid. One possible way to gain additional insights into the age of the *Stowaway *lineages of the Triticeae would be to survey *Stowaway *variation in genera related to, but outside of the tribe. Phylogenetic relationships linking Triticeae elements to multiple lineages outside the tribe would be consistent with shared ancestral polymorphism.

The high level of homoplasy exhibited by the intron 4 *Stowaway *elements is unmistakable, given the phylogenetic context provided by the β-amylase gene tree; there is little correspondence between presence vs. absence of the element and the phylogenetic estimate. In any case, the character states "present" and "absent" have little meaning here because the elements that are present, though found at orthologous sites within the gene, are not themselves related as orthologs, and sites from which the element is absent are variable in terms of sequence. Thus, while the presence vs. absence of a *Stowaway *element has been used for phylogenetic inference in AA-genome species of rice [36,37], the general utility of MITEs as phylogenetic markers at higher taxonomic levels should not be assumed [13]. The specific genetic mechanisms underlying the homoplastic losses and gains cannot be determined from the observed historical patterns alone, but the phylogenetic perspective does reveal the complexity of MITE evolutionary dynamics over time, and complements recent studies documenting ongoing activity of MITEs and *mariner*-like elements.

## Conclusion

*Stowaway *MITEs have undergone multiple deletions from and insertions into the same site in β-amylase intron 4 during the history of the tribe. Multiple losses are supported by sequence variation among the putative sites of element excision/degradation, and the phylogenetic distribution of those sites. Multiple gains are supported by the polyphyletic distribution of five β-amylase elements, and their non-monophyly relative to elements at non-orthologous loci in other Triticeae genomes. The complex history of gains and losses contraindicates the use of either the presence vs. absence of, or sequence comparisons among, these elements for organismal phylogeny reconstruction. While phylogenetic analyses of historical element activity do not allow genetic mechanisms of gain and loss to be inferred, they do provide insights into the long-term dynamics of element evolution. Thus, the historical perspective provided by a phylogenetic approach is complementary to studies of ongoing transposon activity.

## Methods

A set of aligned Triticeae β-amylase sequences [Genbank:AY821686–AY821734] was generated for a phylogenetic study of the tribe [15]; the taxon list with authorities and accession numbers, and the laboratory methods used for obtaining the sequences, are provided therein. Briefly, a 1400-bp portion of the ubiquitously-expressed β-amylase gene [38] was amplified using primers in exon 2 (2a-for, GCCATCATGTCRTTCCACCA) and exon 5 (5a-bac, TCRGCTGCATGGTTTGGAAC) [15], and amplified products were cloned into pGEM-T Easy vectors (Promega, Madison, WI, USA). Cloned products were amplified directly from colonies and cleaned using shrimp alkaline phosphatase and exonuclease I (USB, Cleveland, OH, USA). After cleaning, fragments were sequenced with BigDye Terminator v. 3.1 (Applied Biosystems, Foster City, CA, USA) using the amplification primers and four internal primers (3a-bac, ATGAATTCTCCRAYGCCTGG; 3a-for, CCAGGCRTNGGAGAATTCAT; 4a-bac, CTGCTGCTGCTTTGAARTCTG; and 4b-for, TACCTRSAAGCAGACTTCAAAG) [15]. Sequence alignments were done using Clustal V [39], with some manual adjustments.

Short transposable elements were initially recognized as highly variable insertions with conserved termini in some β-amylase introns. The basic local alignment search tool (BLAST) [40] yielded significant alignments to *Stowaway *elements from barley, wheat, and/or rye in the Entrez nucleotide database [41]. Each of the five β-amylase elements was used for a separate nucleotide-nucleotide BLAST search of all organisms in Entrez, with low complexity filter on, and a word size of 11. BLAST searches can recover high-score (low E-value) matches based solely on short regions of high similarity (including *Stowaway *TIRs), so searches were run twice; the TIR sequences were first included with, and then excluded from, the query sequences. The best matches in terms of length and sequence identity were similar for both sets of searches, and the results based on the full sequences are presented. The β-amylase elements' secondary structures (not shown) were predicted using mfold v. 3.2 [42] on the Rensselaer bioinformatics web server [43].

Phylogenetic distribution of elements was assessed relative to the β-amylase gene tree, which was estimated after exclusion of the *Stowaway *elements themselves. Details of the sampling, analysis methods, and interpretation of the resulting phylogenetic tree are provided elsewhere [15]. In brief, aligned sequences were analyzed with PAUP* 4.0b10 [44] using maximum likelihood (ML) under a general time reversible model of sequence evolution [45] with some sites assumed to be invariable, and with rate variation among the remaining sites assumed to follow a gamma distribution [46,47]. Model parameter optimization, and tree searches using fixed optimized parameters, were done using a successive approximations approach [48]. Bootstrap branch support was estimated using PAUP* 4.0b10 [44] based on 100 ML bootstrap replicates under the same model and model parameters as were used for phylogeny estimation, and Bayesian posterior probability values were obtained using MrBayes 3.0 [49]. The resulting tree is used to illustrate the phylogenetic distribution of *Stowaway *elements and excision sites.

## Competing interests

The author(s) declare that they have no competing interests.
